# The ACE of Hearts Serious Game for Young People With Adverse Childhood Experiences: Experience-Based Co-Design and Agile Development Study

**DOI:** 10.2196/99789

**Published:** 2026-10-05

**Authors:** Haiou Zhu, Natalie Bisal, Minhua Ma, Harsimran Sansoy, Isabelle Butcher, Kamaldeep Bhui

**Affiliations:** 1 CHIMES Collaborative, Department of Psychiatry, University of Oxford, Warneford Hospital, Warneford Lane, Headington, Oxford, OX3 7JX, United Kingdom, +44 1865 618200; 2 Surrey Institute for People-Centred AI, University of Surrey, Guildford, United Kingdom; 3 World Psychiatric Association Collaborating Centre, Oxford, United Kingdom; 4 Wadham College, University of Oxford, Oxford, United Kingdom

**Keywords:** adverse childhood experiences, serious games, experience-based co-design, agile development, digital mental health, adolescents, participatory design

## Abstract

**Background:**

Young people exposed to adverse childhood experiences (ACEs) are at risk of poor mental and physical health across the life course, yet many struggle to access timely help. Serious games offer an engaging, scalable, and stigma-reducing support, but few are co-designed with young people who have lived experience of ACEs, and methodological accounts of how participatory design is operationalized alongside software delivery are scarce.

**Objective:**

We aimed to (1) develop and apply a novel methodological framework integrating experience-based co-design (EBCD) with agile game development to co-design a serious game with young people affected by ACEs, (2) describe the co-designed game prototype and the design decisions shaped by youth participation, and (3) examine the ethical and safeguarding considerations arising from co-designing digital mental health interventions with trauma-exposed young people.

**Methods:**

We conducted a participatory co-design study comprising 12 workshops across 8 iterative co-design sprints, involving 18 diverse young people (aged 12‐24 years) with lived experience of ACEs, 5 professional stakeholders, and a multidisciplinary game development team. Young people were recruited across England through partner youth organizations and regional Young People’s Advisory Groups. Co-design ran from June 2022 to April 2024 in hybrid and online formats. Baseline characteristics include demographics, and mental health and well-being measures. EBCD activities were mapped onto agile practices (sprint planning, sprint reviews, and retrospectives); all feedback was logged in a “you said, we did” form. Analyses were descriptive, and reporting followed the GRIPP2-LF (Guidance for Reporting Involvement of Patients and the Public, long-form) checklist.

**Results:**

The resulting prototype, ACE of Hearts, comprised a central “cozy den” hub and 4 mini-games addressing bereavement and caregiving, trauma and disability, gender dysphoria, and poverty. Youth feedback directly shaped 47 documented feedback and design decisions. Co-design participants (14 of 18 participants) were diverse in gender (8/14, 57% men or boys; 3/14, 21% women or girls; 1/14, 7% nonbinary; 2/14, 14% another identity; and 3/14, 21% transgender), ethnicity (5/14, 36% from minoritized ethnic backgrounds), sexuality (6/14, 43% bisexual or pansexual), and neurodivergence (7/14, 50% autistic), with elevated levels of depression (mean Patient Health Questionnaire 9-item scale 8.8, SD 4.5) and anxiety (mean Generalized Anxiety Disorder 7-item scale 8.4, SD 3.9). Mean posttraumatic stress symptom severity (Children's Revised Impact of Event Scale, 8-item; n=13) was 22.5 (SD 7.7). Design innovations included metaphoric storytelling, integration of narrative exposure therapy principles into gameplay, and an embedded ethics framework addressing representation, agency, and emotional safety.

**Conclusions:**

Integrating EBCD with agile development is a feasible and transparent method for coproducing trauma-informed serious games with young people who have lived experience of ACEs. The approach is transferable to other participatory digital-health projects involving vulnerable populations. Future work would evaluate acceptability, feasibility, and clinical outcomes.

## Introduction

Adverse childhood experiences (ACEs) refer to traumatic or stressful events in childhood, including abuse (emotional, physical, or sexual), neglect, and household challenges such as domestic violence, parental substance misuse, mental illness, incarceration, or separation [1,2]. ACEs tend to cluster in socioeconomically disadvantaged communities and have profound, long-term impacts on health across the lifespan [3,4]. In a meta-analysis of 253,719 participants, individuals with 4 or more ACEs showed elevated odds across 23 mental health and behavioral outcomes, including depression, posttraumatic stress disorder, self-harm, and suicide attempts [5]. Beyond individual suffering, ACEs confer substantial societal costs and are estimated to cost England and Wales £42.8 billion (a currency exchange rate of £1=US $1.35 was applicable) annually [6]. Mental health problems, one consequence of ACEs, cost society around £300 billion a year in 2022 [7]. Child and adolescent services across England are running at capacity with waiting lists, in some places as long as 6 years [8]. Those facing multiple disadvantages and identities of exclusion (by ethnicity, neurodiversity, and LGBTQ+ [lesbian, gay, bisexual, transgender, and queer] status), and/or living in rural or coastal areas experience additional barriers to accessing mental health support [9]. Even when seen in services, young people often fear stigma, harmful consequences, and being ignored or invalidated by practitioners who are increasingly pressed to provide short-term, discrete interventions [10,11]. Reducing waiting lists and providing more care and support at home, away from hospitals, is a global priority for prevention and mental health promotion [12].

Digital technologies and game-based interventions offer a novel avenue to meet this need. Video games are an integral part of youth culture and can deliver interactive experiences in a familiar, appealing format [13]. Serious games, designed for educational or therapeutic purposes beyond entertainment, have emerged as engaging mental health interventions for adolescents [14]. Serious games leverage playful interactions, narrative, and reward systems that are inherently appealing to young people, potentially increasing engagement and adherence to an intervention [15]. Recent meta-reviews have shown positive impacts of game-based interventions on anxiety, depression, and related symptoms in children and adolescents [16,17]. More recent syntheses report promising efficacy for serious games promoting mental health literacy and emotional regulation in young people [18]. Examples include MindLight, a biofeedback game for child anxiety, and SPARX, a fantasy role-playing game for adolescent depression, which demonstrated efficacy comparable to traditional therapies in controlled studies [19,20]. In randomized trials, MindLight reduced anxiety symptoms comparably to online cognitive behavioral therapy [21], and SPARX achieved remission rates superior to usual care among 187 adolescents seeking help for depression [22]. A game-based intervention was recently authorized by the US Food and Drug Administration for pediatric attention-deficit/hyperactivity disorder [23]. Serious games thus represent a novel modality for supporting young people’s mental health, yet few have been designed specifically to address trauma- or adversity-related mental health needs [17].

How such games are designed is as critical as whether they work. Co-design, the meaningful involvement of end users as partners in intervention development, is increasingly recognized as essential for creating digital health innovations that are acceptable, relevant, and effective [24]. Recent reviews have begun to map how digital health technologies might screen for and mitigate the mental health consequences of ACEs in children and young people, while noting that few tools are designed with, rather than merely for, those affected [25]. Another systematic review of 41 co-design studies developing mental health interventions with young people found that one-third were assessed as low quality and that genuine youth participation was limited in two-thirds of studies [26]. To bridge this quality gap, future co-design studies need to prioritize theoretical grounding, methodological rigor, and transparent reporting of participant engagement [27].

This study addresses these gaps in the context of ATTUNE (Understanding Mechanisms and Mental Health Impacts of Adverse Childhood Experiences), an interdisciplinary, arts-science research program funded by UK Research and Innovation. We co-designed and developed ACE of Hearts, a novel serious digital game for young people (aged 12‐24 y) who have experienced childhood adversities. The game is intended as a self-guided, supportive, and educational resource for use in community and home settings, complementary to, and not a substitute for, professional care. It may also be used with facilitation by practitioners in youth, school, and community settings. In particular, systematic accounts of how participatory engagement is operationalized alongside iterative software delivery, and how competing priorities such as youth preference, clinical safety, and technical feasibility are reconciled, remain scarce [28].

Situated within the development phase of the Medical Research Council framework for complex interventions [29], we integrated 2 complementary methodologies: experience-based co-design (EBCD) and agile development. Originating in health service improvement, EBCD centers the lived experiences of service users and systematically translates experiential data into design priorities [30,31]. In parallel, agile development, a software engineering framework characterized by iterative “sprints” with continuous feedback and adaptation [32], is the standard approach for digital product development but has rarely been formally integrated with participatory health research methodologies. The integration of these approaches addresses a fundamental tension in serious game design, the need to balance the deep participatory engagement of EBCD with the iterative technical fluency of agile [33].

In this paper, we present (1) a novel methodological framework integrating EBCD with agile game development, demonstrating how these complementary approaches can be combined; (2) the co-designed game prototype and a transparent audit trail tracing how youth participation shaped substantive design decisions; and (3) critical reflections on ethical safeguarding and the realities of power sharing when co-designing with trauma-exposed young people.

## Methods

### Research Design and Setting

This was a participatory co-design study integrating EBCD with agile software development, with a cross-sectional baseline survey characterizing participants. The method was designed to enable iteration and therefore facilitate deep co-design. This study was conducted within the UK Research and Innovation–funded ATTUNE program. In total, we conducted 12 sessions of co-design, 8 dedicated to general design and 4 to ethical dimensions. We collected qualitative co-design data throughout, supplemented by a baseline survey characterizing the co-design group. The game’s acceptability and preliminary outcomes are evaluated in a separately reported study.

### Overview of the Integrated EBCD-Agile Framework

The ACE of Hearts prototype was developed using an iterative EBCD approach integrated into an agile game development framework structured across 8 development sprints (Figure 1). Each sprint comprised defined goals at its start and evaluation at its end, with findings informing subsequent sprint goals. The 8 sprints of approximately 6 weeks each accounted for approximately 11 months of active development, and the remainder of the 12-month phase encompassed concept refinement, ethics review, participant recruitment, and scheduling around participants’ school or work commitments. Agile practices comprised sprint planning (translating co-design outputs, such as prioritized emotional touchpoints, preference rankings, and storyboards, into sprint goals and a prioritized backlog of user stories), regular development within the game team, sprint reviews (in which young people played builds of the game and gave structured feedback), and retrospectives (in which the research and development teams reviewed process, safeguarding, and priorities). Where youth preferences, clinical safety, technical feasibility, and budget were in tension, clinical safety took precedence, followed by technical feasibility within the funded scope; youth suggestions that could not be implemented were documented in the “you said, we did” form with reasons and revisited where possible. The process was organized into four phases: (1) understanding ACEs, drawing on materials from earlier ATTUNE creative workshops [2]; (2) concept development, exploring game preferences and generating storylines, characters, and game mechanics; (3) game development, with iterative playtesting and feedback driving design refinements; and (4) game evaluation, involving a preliminary feasibility and acceptability study, which is reported separately. A summary of co-design sessions, stakeholders, and activities is provided in Table S1 in Multimedia Appendix 1.

**Figure 1.**
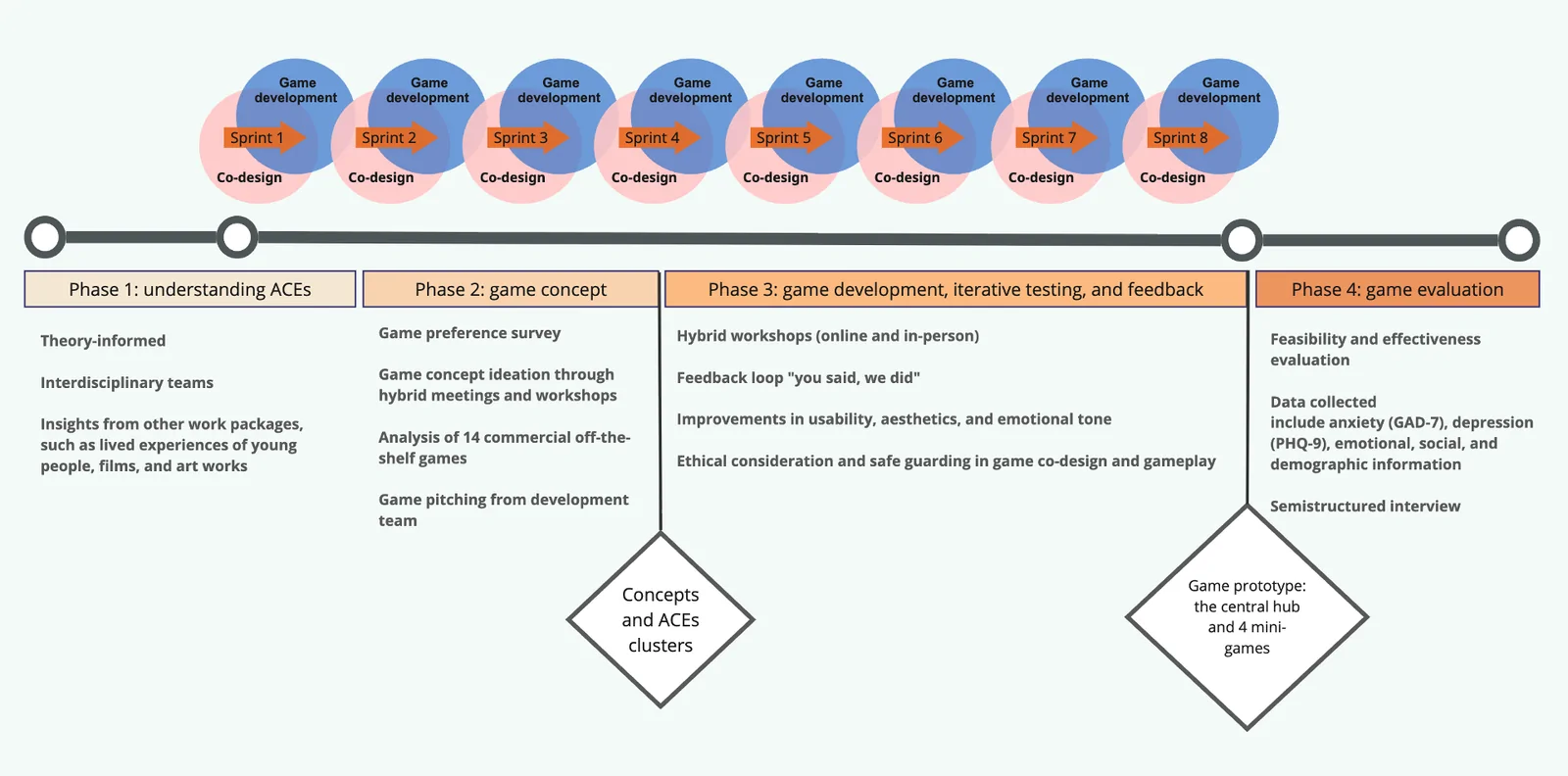
Overview of the integrated experience-based co-design (EBCD) and agile development framework used to co-design the ACE of Hearts serious game with 18 young people (aged 12‐24 y) with lived experience of adverse childhood experiences, United Kingdom, June 2022 to April 2024. ACE: adverse childhood experience; GAD-7: Generalized Anxiety Disorder 7-item scale; PHQ-9: Patient Health Questionnaire 9-item scale.

### Inclusion and Exclusion Criteria

Inclusion criteria for young people were the following: being aged 12‐24 years, self-identified lived experience of ACEs, living in the United Kingdom, and being able to provide informed consent. Young people were not eligible if they were unable to participate safely in group creative activities, if they were currently receiving acute crisis care, or if consent could not be obtained.

### Participant Characteristics

Baseline survey data were available for 14 of the 18 young people (full details in Tables S2 and S3 in Multimedia Appendix 1). The mean age was 19.1 (SD 3.4; range 11‐24) years. One participant was aged 11 years at the time of the baseline survey, which accounts for the observed range of 11‐24 years against the 12‐24 year eligibility criterion. Of 14 respondents, 8 (57%) identified as a man or boy, 3 (21%) as a woman or girl, 1 (7%) as nonbinary, and 2 (14%) with another gender identity; 3 (21%) reported being transgender; 6 (43%) identified as bisexual or pansexual; 6 (43%) were White British and 5 (36%) from Black, mixed, or other ethnic backgrounds; and 7 (50%) reported at least one neurodivergent condition, most commonly autism (7, 50%) and attention-deficit/hyperactivity disorder (4, 29%). Nine (64%) participants lived in urban areas and 5 (36%) participants in rural or coastal areas. Participants presented with elevated mental health difficulties: mean Patient Health Questionnaire 9-item scale (PHQ-9) depression score was 8.8 (SD 4.5), with 7 (50%) participants meeting the threshold for moderate or higher severity; mean Generalized Anxiety Disorder 7-item scale (GAD-7) anxiety score was 8.4 (SD 3.9); mean loneliness (4-item UCLA Loneliness Scale [UCLA-4]) was 9.4 (SD 2.5); and mean PTSD (posttraumatic stress disorder) symptom severity (Children's Revised Impact of Event Scale, 8-item [CRIES-8]; n=13) was 22.5 (SD 7.7).

### Sampling Procedures

The core co-design team brought together a multidisciplinary group comprising 18 young people (aged 12‐24 years) with lived experiences of adversity and diverse backgrounds, 5 adult stakeholders comprising experts in clinical psychology, psychiatry, game studies, and medical ethics, and a game development team consisting of a game writer, a 3D artist, an animator, a programmer, and 2 creative arts practitioners. Co-design activities ran from June 2022 to April 2024 in hybrid and online formats to enable participation by young people across England, including rural and coastal regions. Young people were recruited purposively across the ATTUNE program through partner youth organizations and the regional Young People’s Advisory Groups, with youth workers and organization leads supporting invitations. Adult professional stakeholders and the game development team were recruited by convenience sampling from researchers and professionals working within the program.

### Sample Size, Power, and Precision

A priori power analysis was not performed, and no target sample size was calculated, because this was a participatory co-design study with descriptive aims rather than a hypothesis-testing design. The sample size was pragmatic, aiming for diversity of lived experience and sustained engagement across sprints. To indicate precision, 95% CIs were 8.8 (95% CI 6.2‐11.4) for PHQ-9, 8.4 (95% CI 6.1‐10.7) for GAD-7, 9.4 (95% CI 8.0‐10.8) for UCLA-4, 22.6 (95% CI 20.2‐25.0) for Short Warwick-Edinburgh Mental Well-Being Scale, 50.1 (95% CI 45.0‐55.2) for Difficulties in Emotion Regulation Scale, short form (DERS-SF), and 22.5 (95% CI 17.8‐27.2) for CRIES-8.

### Measures and Covariates

Baseline sociodemographic measures included age, gender identity, transgender status, sexual orientation, ethnicity, neurodivergence, education and employment status, household composition, caregiver education and employment, and area of residence (urban, rural, or coastal). Mental health and well-being measures comprised depressive symptoms, anxiety symptoms, posttraumatic stress symptoms, loneliness, mental well-being, and difficulties in emotion regulation. Game-related measures comprised weekly play time, favorite games, and factors rated as important to the gaming experience, which informed the phase 2 concept development. These measures were used solely to characterize the co-design group and its starting point for design work. They were not treated as outcomes or predictors, and no covariates were specified or adjusted for because no modeling or inferential analysis was undertaken. The substantive study data were the qualitative co-design outputs and the “you said, we did” design decision log.

### Data Collection

Co-design activities took place between June 2022 and April 2024, in online sessions and in hybrid sessions, enabling participation by young people in rural and coastal as well as urban areas. Baseline data and game preference data were collected through the online survey platform Qualtrics. Co-design data comprised facilitator and observer notes, workshop outputs (including collaborative whiteboards and questionnaires), and playtesting feedback captured during and after each session. All actionable feedback was logged in a “you said, we did” form recording the feedback received, the design response, and the rationale where suggestions were not taken forward.

### Quality of Measurements

Sessions were cofacilitated by researchers trained in co-design facilitation, safeguarding, and trauma-informed practice, and at least 2 team members recorded observations independently at each session, providing multiple observations of the same activities. Self-report measures were completed directly by participants through an online survey platform. Formal interrater reliability was not calculated. The categorization of design decisions was reviewed by the wider research team, with disagreements resolved by consensus.

### Instrumentation

All self-report instruments were previously validated measures used in their standard published form: the PHQ-9 for depressive symptoms [34]; the GAD-7 for anxiety symptoms [35]; the CRIES-8 for posttraumatic stress symptoms [36]; the UCLA-4 [37]; the Short Warwick-Edinburgh Mental Well-Being Scale [38]; and the DERS-SF [39]. Each was selected because it is brief, validated for adolescents and young adults, and already in use across the wider ATTUNE program, allowing consistency of measurement.

### Masking

Masking was not applicable and was not attempted.

### Psychometrics

Reliability and validity coefficients were not estimated within this study’s sample. The self-report measures were used only to describe the composition of the co-design group.

### Conditions and Design

Conditions and design were not applicable.

### Data Diagnostics

No participants were excluded after data collection, and no criteria for postdata-collection exclusion were applied. A total of 4 of the 18 (22.2%) enrolled young people did not complete the baseline survey, so baseline characteristics are reported for 14 of 18 (14/18, 77.8%) participants. At the item level, all measures were complete except the CRIES-8 and the DERS-SF, each of which was completed by 13 of 14 respondents (13/14, 92.9%; 1/14, 7.1% missing). Noncompletion reflected scheduling and availability during the survey window rather than refusal or withdrawal, and these young people continued to take part in co-design activities.

### Analytic Strategy

Quantitative baseline data were summarized descriptively, with means and SDs for continuous measures and counts, percentages, and explicit denominators for categorical measures. Qualitative co-design data were analyzed and categorized by design domain (narrative content and emotional tone; game mechanics and engagement; visual, audio, and interface design; ethics, emotional safety, and safeguarding; accessibility; and language and cultural fit) by the first author, with categorization reviewed by the wider research team and disagreements resolved by consensus.

### Ethical Considerations

The project received ethics approval from the University Research Ethics Committee at the University of Oxford (23/WM/0105). At the start of the project, all participants (and guardians for those aged younger than 16 years) provided informed consent. Participation in any session or activity remained voluntary, and participants could decline or withdraw at any time without giving a reason. All study data were pseudonymized and stored on secure, access-controlled university systems in accordance with UK General Data Protection Regulation requirements. Participants’ characteristics and quotations reported here are deidentified, and no information enabling identification of individual participants is presented. No images of individual participants or users appear in this paper or in the supplementary material. All in-game images are original artwork depicting fictional characters, and no photographs or other identifiable images of participants were used for publication. Young people received £15 per hour as reimbursement for their contribution. In addition to formal ethics approval, our method adopted an embedded ethics approach to game design, whereby ethically relevant issues were assessed and discussed with stakeholders throughout the co-design process, with the game adjusted to ensure responsible innovation. This included issues of representativeness, accessibility, emotional safety, responsible motivation, and responsible mental health and ACEs content.

Given the elevated levels of depression, anxiety, and posttraumatic stress symptoms in the sample, safeguarding was planned into every session. Sessions were cofacilitated by researchers trained in safeguarding and trauma-informed practice, with a named safeguarding lead and an agreed distress protocol. Sessions began with ground rules co-developed with young people. Participants could pause, step out, or leave at any time without explanation, and creative activities were designed so that disclosure of personal trauma was never required. A youth worker or known professional from the partner organization was present or on call for sessions. Any participant showing or reporting distress was offered immediate support and a follow-up check-in and, where indicated, signposting or referral to local services. Escalation pathways to the clinical members of the team (including consultant psychiatrists and clinical psychologists) were available where needed, and sessions ended with a structured check-out and signposting to support resources. No adverse events were recorded during the co-design program.

### Reporting Standards

We report patient and public involvement in accordance with the GRIPP2-LF (Guidance for Reporting Involvement of Patients and the Public, long form) checklist [40], given that co-design with young people with lived experience of ACEs is the primary focus of this study. We also draw on the adolescent and youth engagement reporting checklist to structure our account of the co-design process [41]. Young people were involved as co-designers throughout all phases of the research, from conceptualization through to game refinement.

## Results

### Overview

We present the results in 4 sections: the participant flow, the co-design process, and how youth participation shaped the game (phases 1‐3), the resulting game prototype and its design innovations, and the ethical and safeguarding considerations that emerged.

### Participant Flow

Figure 2 summarizes participant flow through this study: young people were invited through partner organizations and Young People’s Advisory Groups; 18 enrolled as co-designers; 14 completed the baseline survey (4 young people did not, owing to scheduling and availability rather than refusal); and 6‐13 young people participated per session across the 4 phases, alongside 5 adult professional stakeholders.

**Figure 2.**
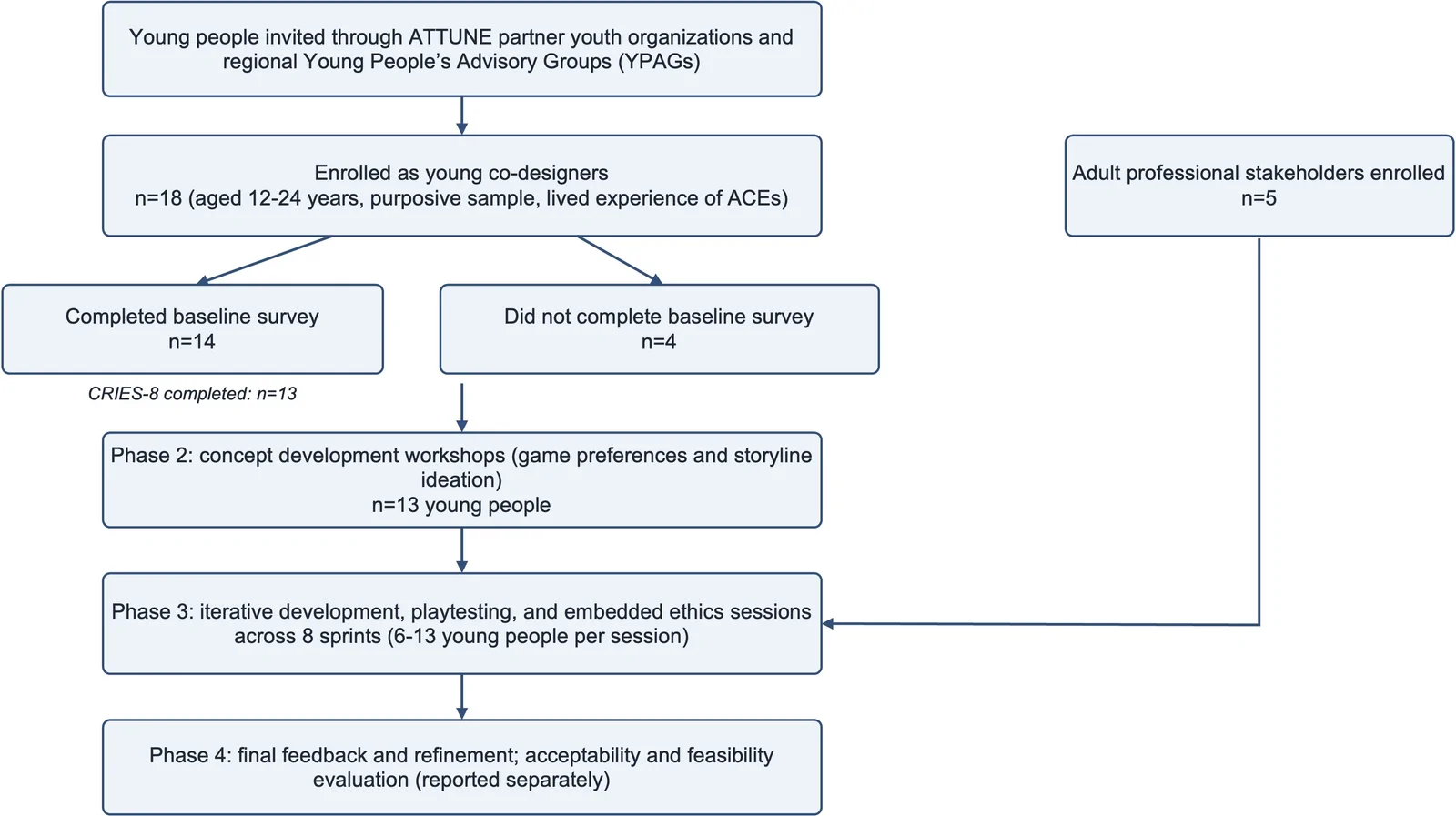
Participant flow through the ACE of Hearts co-design study with young people (aged 12‐24 y) with lived experience of adverse childhood experiences (ACEs), United Kingdom, 2022‐2024. ACE: adverse childhood experience; ATTUNE: Understanding Mechanisms and Mental Health Impacts of Adverse Childhood Experiences; CRIES-8: Children's Revised Impact of Event Scale, 8-item; YPAG: Young People’s Advisory Group.

### Phase 1: Understanding ACEs

This study was informed by earlier phases (corresponding to work packages 1‐2) of the program, which explored the psychological and geo-socioeconomic mechanisms through which ACEs affect the mental health and lives of young people [42,43]. These studies used creative and participatory arts-based approaches to generate new, transformative experiential data, deepening understanding of key mechanisms and informing action for prevention and care. The outputs included resources such as magazines, short animations, and films, and neurodiversity considerations. These materials provided the research team with experiential understanding of ACEs from young people’s perspectives, and they became direct inputs to game content. For example, the magazines became the digital magazine in the Cozy Den, while real-life accounts of young people’s adverse experiences in accessing mental health services were incorporated into the dial it back mini-game.

Building on this foundation, a multidisciplinary research team comprising specialists in serious games, clinical psychology, psychiatry, and medical ethics conducted a game analysis study, playing and reviewing 14 commercial off-the-shelf games [44]. Using an autoethnographic approach, the team analyzed features and mechanics while reflecting on the player’s role in meaning-making. These reflections informed the selection of game concepts and ACE-related themes for the development phase.

### Phase 2: Game Concept Development

Early concept ideation activities explored game preferences and playing experiences related to mental health with 13 young people (Figure S1 in Multimedia Appendix 1). The most frequently named were Minecraft (6 mentions) and Apex Legends (5 mentions), followed by GTA 5 and Subway Surfers (4 mentions each). Most participants were unable to name any games that depicted mental health experiences. Participants found a variety of factors important for their playing experience, summarized as ranked frequencies. Excitement and playing with friends were rated most highly on average (mean 8.6 and 8.5 of 10), followed by engaging with a good story (mean 7.7) and taking time out (mean 7.5). Getting a high score (mean 6.4) and being part of a game community (mean 4.3) were rated lower and showed the greatest variation, with several participants rating them as unimportant (0-1) while others rated them maximally important (10). A recurring motif was that game worlds provide spaces of retreat from “the real world,” offering relief and a sense of ownership, an insight that directly shaped the design of the cozy den hub as a safe, customizable home base.

Insights from phase 1 indicated several priority themes, including family loss, financial hardship, and disability, which became candidates for game storylines. During concept ideation, some participants emphasized the challenges of gender identity struggles and the lack of representation of transgender youth in mental health media. In response, the team agreed to develop a mini-game on this theme, which emerged unexpectedly from the workshops but filled an important gap identified by the young co-designers.

### Phase 3: Game Development—Iterative Development, Play Testing, and Feedback Loops

Phase 3 encompassed the main development of the game prototype and multiple cycles of play testing with feedback. Participants played early builds of each mini-game and the hub, and provided detailed feedback on enjoyment, emotional impact, clarity, and areas for improvement. Their feedback directly drove numerous design refinements. Table 1 presents an overview of key user feedback and the corresponding design actions taken. The full “you said, we did” table is provided in Table S4 in Multimedia Appendix 1.

**Table 1.** Selected examples of youth feedback and corresponding design decisions in the ACE of Hearts co-design study with young people (aged 12‐24 y) with lived experience of adverse childhood experiences, United Kingdom, 2022‐2024.

Mini-game	Domain	Key feedback	Design action
Horse and foal	Narrative pacing and emotional tone	“Shift to loss was too dramatic at the end”; “ending quite jarring but I think that helped to convey the loss”	Additional grief-processing chapter created; foreshadowing implemented; supportive messages and signposting added
Horse and foal	Visual design	Color palette described as too dark and cold	Revised palette with brighter seasonal backgrounds (spring/summer)
Dial it back	Narrative	Concern that visible physical trauma overshadowed less visible ACEs^a^; home environment “too big and tidy”	Clarified narrative to explicitly acknowledge multiple ACEs including neurodiversity; adjusted visuals for realism
Out of my shell	Metaphor	“I love the metaphor, an inventive way preventing themes from being too upsetting”; concerns about dragon misinterpretation	Expanded dragon metaphor with explicit narrative clarification; added breathing exercise mechanic and guardian NPCs^b^
Hard times	Realism	“Worried about trivialization” of poverty; requested systemic framing (“unfair system designed to keep people within margins”)	Multiple endings implemented; reinforced message that systemic factors cause poverty; added practical support signposting
General	Cultural fit	Confusion about US cultural references (Kansas); colloquialisms felt “awkward”	Replaced references with universally recognizable content; reduced colloquialisms
General	Character	“Ordinary people can have mental health problems, not just green-haired weirdos”	Removed negative connotations from character descriptions; diversified representation across mini-games

a ACE: adverse childhood experience.

b NPC: nonplayer character.

Considered in aggregate, the 47 logged decisions were distributed across 6 design domains: narrative content and emotional tone (n=27); game features, visuals, and engagement (n=8); ethics and safeguarding (n=7); accessibility (n=2); and language and cultural fit (n=3). A small number of suggestions (n=3) were not taken forward within the current prototype, most commonly because of technical feasibility or budget constraints (eg, multiplayer play) or scope; each is documented with its rationale in Table S4 in Multimedia Appendix 1 and was noted for future development.

Analysis of the documented design decisions reveals 3 patterns in how youth participation shaped the game. First, young people consistently pushed for emotional authenticity tempered by hope, acknowledging that “bad things happen and feelings such as anger or sadness are valid,” while also reassuring players that “you’re not alone and things can get better.” This tension between realism and reassurance drove major narrative restructuring, including the addition of a grief-processing chapter in horse and foal and multiple endings in hard times. Second, participants articulated a sophisticated preference for metaphorical rather than literal representations of trauma, consistently endorsing approaches that created enough proximity to validate experience, but enough abstraction to prevent retraumatization. Third, youth feedback frequently identified cultural assumptions embedded in the game content (eg, US cultural references and colloquialisms that felt inauthentic) that the adult design team had not recognized, demonstrating the irreplaceable value of genuine participatory design.

### The ACE of Hearts Game Prototype

#### Overview

The resulting prototype (Figure 3) comprises a central hub space, the “cozy den,” which connects 4 fully developed mini-games, addressing adversity-related themes: bereavement and caring for a younger sibling (horse and foal), financial hardship (hard times), living with disability and the aftermath of traumatic injury (dial it back), and the stigma, bullying, and lack of representation experienced by young people with gender dysphoria (out of my shell). Game details are presented in another publication [45]. The present paper is distinct in reporting the co-design methodology, the safeguarding and embedded ethics processes, and the game design decisions. The cozy den itself functions as a persistent, customizable home base: players personalize their virtual bedroom (eg, changing posters and music), interact with objects (eg, playing the guitar, feeding the fish, drawing, and reading the digital magazine derived from the ATTUNE magazines), and unlock each mini-game through the in-game phone and computer, returning to the den between and after mini-games as a space for decompression and safety.

**Figure 3.**
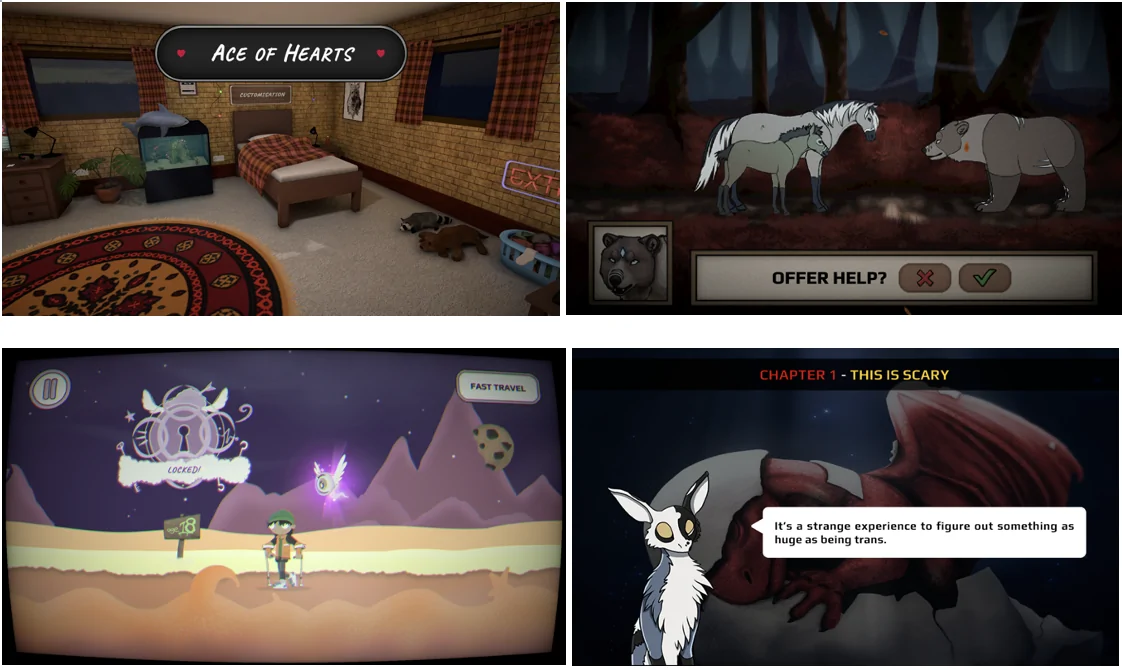
Screenshots of the ACE of Hearts game. Panels show the cozy den hub and the horse and foal, dial it back, and out of my shell mini-games. The game was co-designed with young people (aged 12‐24 y) with lived experience of adverse childhood experiences, United Kingdom, 2022‐2024. No identifiable individuals are shown; all artwork is original and depicts fictional characters.

#### Horse and Foal

This 2D side-scrolling narrative mini-game explores themes of sibling caregiving and bereavement. The player controls Carla, a young horse, who must take care of her little sister (a foal named Rhea) as they journey through pastoral landscapes symbolizing the progression of Rhea’s illness and eventual loss. In early testing, youth players praised the use of animal characters and seasons but found the initial pacing too dark or abrupt. In response, the visuals were brightened, the chapter sequence was reordered to begin in spring, and an epilogue scene was added showing Carla remembering her sister but continuing forward (the final choice: “keep moving on”).

#### Dial It Back

Inspired by narrative exposure therapy (NET) principles [46], this mini-game unfolds an interactive story about confronting memories. The protagonist, Oli, a 16-year-old boy who has experienced multiple adverse events and physical disability, is presented in the third person, a deliberate choice based on co-design feedback that some distance can make processing trauma safer. The player helps Oli reconstruct his life story by identifying significant memories and placing a flower or a stone to mark each as good or bad, drawing directly from NET’s life-line technique. During playtests, a journal feature was added, and the narrative was adjusted so that Oli chooses to seek counseling, framing help-seeking as a positive resolution.

#### Out of My Shell

This tower-defense mini-game uses a fantasy metaphor to explore identity, stigma, and finding one’s true self, drawing on experiences of gender dysphoria and bullying. The protagonist, Flo, feels like a “dragon in disguise,” a metaphor for having a hidden true identity. Flo must defend their “nest” (representing a sense of self) from “enemies” symbolizing bullies, rumors, and negative self-talk. Feedback led to a guided breathing exercise mechanic and 2 guardian nonplayer characters providing supportive messages. By the final wave, Flo can transform fully into a dragon, signaling resilience and self-acceptance. The dragon metaphor and the portrayal of Flo’s experiences were reviewed with co-designers with relevant lived experience, including 1 game artist and 3 transgender participants, whose feedback shaped explicit dialogue acknowledging the diversity of transgender experiences, including a nuanced, nonstigmatizing treatment of detransition.

#### Hard Times

This 3D first-person scenario addresses financial hardship and family stress. The player steps into the shoes of Mina, a teenager struggling to pay rent in a small London flat. The core mechanic is managing scarce resources by making tough choices. Youth co-designers commented on how immersive and relatable the scenario felt but found the original eviction ending bleak. In response, multiple endings were implemented, ranging from hopeful (the family obtains assistance) to bittersweet (they move, but relationships strengthen), ensuring players retain agency and hope.

### Design Innovations

#### Overview

Beyond the individual mini-games, 3 cross-cutting design innovations emerged from the co-design process that represent this study’s contribution to serious game design methodology for trauma-exposed populations.

#### The Use of Metaphor and Multimodal Storytelling

A defining feature of ACE of Hearts is the systematic use of metaphor and multimodal storytelling. From the earliest design stages, we recognized that directly depicting real traumatic events might be triggering for some young players. Instead, inspired by techniques used in art therapy, we created a symbolic layer for each narrative. This design choice is grounded in therapeutic theory, which suggests that metaphors enable individuals to process trauma and express emotions indirectly [47,48]. Video games are particularly well-suited to convey such metaphors because they operate across visual, auditory, and interactive channels, creating an “ecosystem of meaning-making” [49]. Crucially, the metaphorical approach was not imposed by the design team but validated and refined by the youth co-designers, who consistently endorsed it as “an inventive way preventing themes from being too upsetting.” A summary of the multimodal metaphor design is provided in Table S5 in Multimedia Appendix 1.

#### Drawing on NET

Another salient and critical design innovation is the integration of therapeutic techniques, specifically NET, in dial it back. NET is a short-term therapy in which individuals tell their trauma stories through unfolding memories within a biographical context [46]. Core elements of NET in the dial it back mini-game include the construction of a “lifeline” of events and recounting details of each traumatic memory. We worked closely with clinical advisors, including a psychiatrist experienced in NET, to gamify these elements. The game’s memory constellation mechanic and lifeline timeline mirror the process of NET, engaging the player in the process of organizing life events. Another NET principle implemented was the emphasis on sensory cues: players unlock each memory by moving an “eyeglass” around a stylized brain to find 4 sensory details (smell, sound, touch, and an emotion) associated with that event [50,51]. We drew from narrative exposure therapy for children (KIDNET), the child adaptation of NET, which suggests using more visual aids and metaphors for younger audiences [52]. While dial it back incorporates elements of NET, it is important to note that it is not designed to serve as a standalone trauma therapy. Rather, the aim is to create a meaningful and supportive experience that may empower players, promote a sense of control, and encourage help-seeking.

### Embedded Ethics Framework

Designing a mental health game for young people who have experienced distress or adversity required careful attention to ethical and safeguarding considerations throughout development [53]. Rather than treating ethics as a one-off review, we adopted an embedded ethics approach in which ethically relevant issues were assessed and discussed with stakeholders in 4 dedicated co-design sessions. By embedded ethics, we mean the continuous, in-process identification, discussion, and resolution of ethical issues during design, rather than a one-off ethics review. Before the co-design sessions, our research team, comprising clinical and ethics expertise, conducted a systematic review and critical appraisal of the ethical implications of digital gaming interventions for mental health [54]. Based on the findings, we provided comprehensive ethical considerations and related questions in the protocol, structured around 7 domains: representation, accessibility, agency, privacy, emotional safety, positive motivation, and responsible content. In practice, ethical concerns raised by any participant or team member in workshops or playtesting were logged as flagged entries in the design decision, discussed at dedicated embedded ethics review sessions. Table 2 summarizes the ethical dimensions, youth feedback, and actions taken (full details in Table S6 in Multimedia Appendix 1).

**Table 2.** Ethical dimensions, youth feedback, and safeguarding actions in the ACE of Hearts co-design study with young people (aged 12‐24 y) with lived experience of adverse childhood experiences, United Kingdom, 2022‐2024.

Ethical domain	Key concerns raised	Actions taken
Representation	Concerns about stereotyping; requests for ethnic/cultural markers; diverse transgender experiences	Diversified characters across games; culturally accurate elements in narratives; nuanced portrayal of gender identity experiences
Accessibility	Older devices excluded; small text for those who are visually impaired	Compatibility with older devices (iOS and Android); improved font sizes
Agency	Frustration when outcomes could not be influenced by choices; options to personalize or have visible impact	Personalization options in cozy den; clarified that some narratives reflect uncontrollable life events with explicit reassurance
Privacy and transparency	Youth asked about data handling and requested clear communication about development, purpose, and commercial interests; clear indication needed about the limits of the game’s therapeutic value	Explicit in-game information about data use; clear communication about purpose
Emotional safety	Abrupt emotional content; need for external signposting and reporting mechanisms	Extended epilogues; foreshadowing; supportive dialogs; in-game resources and links to support organizations
Positive motivation	Suggested self-care and playtime reminders	Integrated self-care reminders and mindfulness exercises in-game
Responsible content	Concerns about trivialization or harmful portrayals of ACEs^a^ and mental health (poverty, dysphoria, trauma)	Revised narratives and dialogues to responsibly portray mental health and ACEs; avoided stereotypes, and used diverse, respectful representations

a ACE: adverse childhood experience.

It was ethically important that the game’s content respectfully represents the diverse experiences of young people with ACEs, who live in a range of geographic regions of England. In our co-design, we made conscious efforts to include youth collaborators of various identities (including diversity in ethnicity, gender identity, sexual orientation, neurodiversity, and region) so that the ideas generated reflected a broad spectrum of perspectives. This fed directly into the game narratives: each of the 4 mini-games features a distinct protagonist facing distinct adversities, intentionally broadening representation. Accessibility covered both technical accessibility and content accessibility, that is, the comprehensibility of narratives, metaphors, and visuals for diverse young players, including neurodivergent players. Key adjustments included providing compatibility with older and low-specification devices, increasing font sizes, and improving signposting for navigation and storylines.

Promoting a sense of agency and control for young players was central both in our co-design methodology and within the game itself. During the co-design sessions, young people were given real influence over decisions. Within the game, we designed the gameplay to give players meaningful choices and multiple pathways wherever feasible. Within our study, emotional safety refers to presenting emotionally challenging content in ways that validate experience while minimizing the risk of distress or retraumatization. Responsible and positive motivation refers to encouraging engagement without fostering overuse or dependence. Given the potential of the game to trigger distressing feelings, the realism of games can present a double-edged sword, in that it may increase engagement but also risks intensifying emotional responses or even retraumatizing young people [55]. The games were designed to balance the depiction of traumatic experiences enough to validate players’ lived experiences while also incorporating positive messages, supportive characters, and moments of relief. Techniques such as gentle pacing, interactive coping activities (eg, breathing exercises in out of my shell and the “keep moving forward” option in horse and foal), and in-game resources and signposting to sources of real-world support were used to mitigate potential emotional burdens.

## Discussion

### Principal Findings

In this study, we combined EBCD and agile development to develop the ACE of Hearts game, one of the few serious games co-designed specifically with and for young people affected by ACEs. The integrated EBCD-agile framework produced a game prototype that was substantively shaped by the lived experiences and preferences of young people across 8 iterative sprints. Three principal findings emerge from this work. These findings correspond directly to the 3 objectives stated at the end of the Introduction section: the methodological framework, the co-designed prototype and feedback loop, and the ethical and safeguarding considerations.

First, the integration of EBCD with agile development proved both applicable and productive for serious game development with potentially vulnerable populations. EBCD’s emphasis on foregrounding lived experience complemented agile’s capacity for rapid iteration, creating a development process in which youth feedback could be gathered, discussed, and incorporated within sprint cycles. Systematic reviews of EBCD in health care and of co-design in digital health have noted that participatory approaches are rarely coupled to iterative development, while agile-user experience hybrids seldom address power sharing or lived experience [27,28,56]. The resulting framework may serve as a transferable, well-documented model for future digital mental health innovation, whose replicability should be tested in other settings [57].

Second, young people’s contributions substantively shaped the game’s content, mechanics, and ethical safeguards. The transparent feedback and design decisions demonstrate how youth feedback led to concrete design changes, ranging from narrative restructuring (eg, the addition of a grief-processing chapter in horse and foal) to the emergence of entirely new game modules (eg, out of my shell, addressing gender dysphoria, which emerged from co-design discussions rather than the original research plan). This level of traceable participation addresses concerns raised in recent systematic reviews about the quality and depth of youth involvement in co-designed mental health interventions [26].

Third, the use of metaphor and multimodal storytelling, combined with therapeutic techniques adapted from NET, represents a novel approach to serious game design for young people with ACEs. The metaphorical framing—animal characters for bereavement, dragons for gender identity, and sensory constellations for memory processing—was not imposed by the design team but coconstructed with young people, who consistently advocated for approaches that were “real but not overwhelming.” This aligns with therapeutic theory suggesting that metaphors can provide a “safe distance” for exploring difficult themes [44].

### Comparison With Prior Work

This work extends the existing literature in several ways. While prior studies have described co-design of gamified mental health tools [33], few have formally integrated EBCD with agile methods. We do not claim primacy for the game itself: established serious games such as SPARX for adolescent depression [22] and MindLight for childhood anxiety [21], alongside scholarly analyses of commercial autobiographical titles addressing illness and grief, such as That Dragon, Cancer [44,49], demonstrate a maturing field; the contribution of this study is primarily methodological, namely the documented EBCD-agile integration and the transparent design decision audit trail. For example, a framework provides a 5-step model for theory-driven serious game development but does not incorporate EBCD’s emphasis on lived experience [58]. The EBCD-agile synthesis also sits alongside adjacent design methodologies. Lean user experience combines agile cadence with user-experience research through rapid build-measure-learn loops but is commercially oriented and does not center lived experience or power sharing [56]. The Design Council’s Double Diamond structures innovation through discover-define-develop-deliver phases, paralleling EBCD’s discovery-to-co-design arc, but it is not intrinsically trauma-informed and specifies little about how to collaborate with vulnerable users [59]. Our framework differs in combining EBCD’s experiential, touchpoint-based methods and explicit power-sharing commitments with agile’s sprint cadence, while embedding clinical safeguarding and ethics review within the development loop itself. This current paper is also distinct from, and complementary to, 2 related outputs: a short demonstration paper describing the ACE of Hearts game artifact itself [45] and the separately reported study evaluating the game's acceptability and feasibility. The original contributions here are the integrated framework, the safeguarding and embedded ethics processes, and the aggregate analysis of the co-design feedback and game design decisions. Our work also contributes to the growing discourse on reporting quality in co-design research. By adopting the GRIPP2-LF checklist [40] and maintaining transparent communication of design decisions, we address concerns raised in the literature about the variable fidelity of EBCD adaptations and the quality of youth involvement reporting identified in recent systematic reviews [25-27].

Situating the prototype within the serious games literature clarifies the novelty of our game addressing ACEs. Systematic reviews of serious games for youth mental health report promising effects on mental health literacy, emotional regulation, and symptom outcomes, and consistently identify co-design and customization to diverse users as features associated with success [18-20]. Most established studies, however, target a diagnosis or symptom cluster, such as depression in SPARX [22] or anxiety in MindLight [21], rather than the experiences of adversity that cut across diagnoses. Games addressing childhood adversity directly remain uncommon, and where they exist, they have typically focused on prevention or disclosure, for example in child sexual abuse prevention [55], rather than on supporting young people already living with the consequences of ACEs. ACE of Hearts is distinctive in focusing on adversity-related themes nominated by young people themselves, and in treating those themes metaphorically rather than didactically.

Finally, our approach connects the serious games literature to trauma-informed and embedded ethics practice. Trauma-informed design has emerged as a distinct concern in human-computer interaction, emphasizing safety, choice, and control as design properties rather than as procedural safeguards [60]. A scoping review of ethical aspects of gamified mental health interventions for young people found that ethics is commonly interpreted narrowly as research ethics review, that co-design is frequently framed in instrumental terms, and that vulnerability is addressed pragmatically rather than systematically [61]. Work on participatory arts methods with young people affected by ACEs has raised parallel concerns about consent, representation, and emotional safety [53,54]. By running dedicated embedded ethics sessions inside the sprint cycle, and by documenting the resulting safeguarding decisions, our framework treats ethics as ongoing design work rather than as a gate passed before development begins, which is the practice these reviews call for.

### Reflections on Youth Participation

Reflection on the limitations of participation is essential for advancing co-design methodology. The research and facilitation team brought perspectives from serious games and human-computer interaction, clinical psychology, psychiatry, medical ethics, and creative arts practice. We acknowledge that these disciplinary lenses, and the inherent power asymmetry between adult researchers and young co-designers, shaped facilitation and interpretation. While young people were engaged as equal partners, power asymmetries inevitably remained. Such asymmetries are widely recognized in participatory work with young people, where involvement can slide toward tokenism unless decision-making influence is made explicit and visible [26]. The research team held decision-making authority over technical feasibility, budget allocation, and clinical safety considerations. Some design suggestions from young people (eg, multiplayer cooperative play) were noted for future development but could not be implemented within the current prototype due to resource constraints. We sought to mitigate these asymmetries through transparent communication about what was and was not feasible, and by documenting young people’s rationales for suggestions that were not taken forward and revisiting them where possible.

Practical constraints also shaped the co-design process in ways that required ongoing negotiation. Most young participants were in school or college, meaning sessions had to be scheduled after school time to accommodate their availability. We conducted the majority of sessions online to enable participation from young people across England, enabling those in rural or coastal areas to contribute on equal terms. When it was necessary to bring young people and adult professionals together, we designed an accelerated EBCD day held online, in which participants were able to join and leave according to the specific mini-game under discussion as well as their own time constraints. This format was intensive but effective in maximizing reach, allowing us to gather feedback from a larger and more diverse group than a single in-person workshop would have permitted. Flexible and remote formats have similarly been reported to widen reach in co-design with young people, although they require deliberate attention to digital access and to sustaining engagement [57].

### Implications

For research and development teams, the practical implication is that meaningful participation and iterative delivery can be run together, as work on agile-user experience integration and on participatory design in serious games has shown [33,56]. The transferable components of our study are concrete: co-design touchpoints tied to fixed sprint boundaries, a standing design decision log that records rejected as well as accepted suggestions, an explicit priority order when youth preference, clinical safety, technical feasibility, and ethics sessions are scheduled inside the development cycle. Co-design of this intensity requires facilitation time, payment for young people, safeguarding capacity, and practical constraints that are among the reasons co-design is often curtailed or reduced to consultation [26,28]. Where reporting standards for public involvement and for youth engagement increasingly expect demonstrable lived-experience involvement [40,41], an auditable “you said, we did” decision log also provides evidence that involvement shaped the product.

For services and practitioners, ACE of Hearts is designed as a self-guided resource for community and home use that can also be facilitated by a practitioner in youth, school, or community settings. Its plausible near-term roles are supportive rather than therapeutic, offering something to engage with while waiting for services, opening conversations about difficult experiences in a lower-stakes register, and modeling help-seeking through in-game signposting. Realizing any of this depends on implementation rather than on the artifact alone. Syntheses of real-world youth digital mental health delivery consistently find that embedding tools in existing services, sustaining engagement, and securing staff time and workflow fit are harder than building the technology [57]. Practitioners using the game with young people affected by ACEs would need clear guidance on the boundary between self-reflection and therapy [62,63].

### Limitations

This work has several limitations. First, the current mini-games cover 4 ACE-related themes but do not encompass the full spectrum of adversities that young people experience. Second, our co-design participants, while diverse, were a relatively small group (N=18); thus, the narratives and characters may not fully capture the breadth of cultural backgrounds or individual differences [28]. Survey data were available for 14 of 18 participants, limiting the completeness of participant characterization. Third, there are potential risks associated with limited direct professional oversight in a standalone game. While in-game signposting to help-seeking was provided, we recognize that some players may require forms of support beyond the scope of the game. Future iterations should expand to include more adversities, involve a broader range of youth perspectives, and evaluate clinical and cost-effectiveness outcomes. Fourth, this study was conducted in England; the transferability of the game content and of the co-design framework to other cultural and linguistic contexts, age groups, health systems, and forms of adversity is untested. Finally, co-design preferences, however carefully documented, do not in themselves establish the safety, acceptability, or therapeutic value of the game for a wider population [60]. These require separately reported evaluation and future controlled studies.

### Conclusions

In this paper, we presented the design and development of ACE of Hearts, a novel serious game that addresses ACEs through gameplay. By integrating EBCD with agile development and grounding the design in extensive co-design with young people, we created a prototype tailored to the perspectives and experiences of its target audience. The integrated EBCD-agile framework offers a transferable methodological model for future digital mental health innovation with vulnerable populations; its replicability should be tested in other settings. The transparent design decision audit trail demonstrates that meaningful youth participation can substantively shape serious game content, mechanics, and safeguards. The contribution of this study is primarily methodological, namely the documented EBCD-agile integration, within which the embedded ethics approach and the co-design feedback loop are design innovations. The resulting prototype illustrates how serious games may support awareness, self-reflection, and help-seeking, though its feasibility and effectiveness need further research. The game is not intended to replace professional care but to complement existing support systems by empowering young players to explore their experiences and learn coping strategies in a safe, playful environment. We hope that the insights gained from this project will inform the future research and development of serious games for vulnerable populations, ultimately contributing to more creative and empathetic forms of mental health support for young people.

## Supplementary material

10.2196/99789 Multimedia Appendix 1 Tables and figures showing participant characteristics, the co-design details, and ethical considerations.

## Abbreviations

ACE: adverse childhood experience
ATTUNE: Understanding Mechanisms and Mental Health Impacts of Adverse Childhood Experiences
CRIES-8: Children's Revised Impact of Event Scale, 8-item
DERS-SF: Difficulties in Emotion Regulation Scale, short form
EBCD: experience-based co-design
GAD-7: Generalized Anxiety Disorder 7-item scale
GRIPP2-LF: Guidance for Reporting Involvement of Patients and the Public, long form
KIDNET: narrative exposure therapy for children
LGBTQ: lesbian, gay, bisexual, transgender, and queer
NET: narrative exposure therapy
PHQ-9: Patient Health Questionnaire 9-item scale
PTSD: posttraumatic stress disorder
UCLA-4: 4-item UCLA Loneliness Scale

## References

[1] Zhou Y, Kivimäki M, Holt-Lunstad J (2025). Stressful life events in childhood and adulthood and risk of physical, psychological and cognitive multimorbidities: a multicohort study. eClinicalMedicine.

[2] Bhui K, Butcher I (2023). The trouble with trauma and triggering. Lancet Psychiatry.

[3] van Woerden H The Annual Report of the Director of Public Health 2018: Adverse Childhood Experiences, Resilience and Trauma Informed Care — A Public Health Approach to Understanding and Responding to Adversity. https://www.nhshighland.scot.nhs.uk/about/publications-and-public-records/director-of-public-health-annual-report/.

[4] Meeker EC, O’Connor BC, Kelly LM, Hodgeman DD, Scheel-Jones AH, Berbary C (2021). The impact of adverse childhood experiences on adolescent health risk indicators in a community sample. Psychol Trauma.

[5] Hughes K, Bellis MA, Hardcastle KA (2017). The effect of multiple adverse childhood experiences on health: a systematic review and meta-analysis. Lancet Public Health.

[6] Hughes K, Ford K, Kadel R, Sharp CA, Bellis MA (2020). Health and financial burden of adverse childhood experiences in England and Wales: a combined primary data study of five surveys. BMJ Open.

[7] Cardoso F, McHayle Z (2024). The economic and social costs of mental ill health. Centre for Mental Health.

[8] Mental health pressures in england. British Medical Association.

[9] (2021). Chief Medical Officer’s Annual Report 2021 - Health in Coastal Communities. https://assets.publishing.service.gov.uk/media/60f98750e90e0703bbd94a41/cmo-annual_report-2021-health-in-coastal-communities-accessible.pdf.

[10] White SR, Soneson E, Fazel M, OxWell Study Team (2024). Networks of care for the modern adolescent. Psychol Med.

[11] Zhao R, Amanvermez Y, Pei J (2025). Research review: help‐seeking intentions, behaviors, and barriers in college students – a systematic review and meta‐analysis. Child Psychol Psychiatry.

[12] Baird S, Choonara S, Azzopardi PS (2025). A call to action: the second Lancet Commission on adolescent health and wellbeing. Lancet.

[13] Vajawat B, Varshney P, Banerjee D (2021). Digital gaming interventions in psychiatry: evidence, applications and challenges. Psychiatry Res.

[14] Torous J, Bucci S, Bell IH (2021). The growing field of digital psychiatry: current evidence and the future of apps, social media, chatbots, and virtual reality. World Psychiatry.

[15] Fleming T, Poppelaars M, Thabrew H (2023). The role of gamification in digital mental health. World Psychiatry.

[16] Wols A, Pingel M, Lichtwarck-Aschoff A, Granic I (2024). Effectiveness of applied and casual games for young people’s mental health: a systematic review of randomised controlled studies. Clin Psychol Rev.

[17] Eckardt JP (2024). Therapeutic uses of gaming in mental health: an untapped potential. JMIR Serious Games.

[18] Zeiler M, Vögl S, Prinz U (2025). Game design, effectiveness, and implementation of serious games promoting aspects of mental health literacy among children and adolescents: systematic review. JMIR Ment Health.

[19] Tsui TYL, DeFrance K, Khalid-Khan S, Granic I, Hollenstein T (2020). Reductions of anxiety symptoms, state anxiety, and anxious arousal in youth playing the videogame MindLight compared to online cognitive behavioral therapy. Games Health J.

[20] Schoneveld EA, Wols A, Lichtwarck-Aschoff A, Otten R, Granic I (2020). Mental health outcomes of an applied game for children with elevated anxiety symptoms: a randomized controlled non-inferiority trial. J Child Fam Stud.

[21] Schoneveld EA, Malmberg M, Lichtwarck-Aschoff A, Verheijen GP, Engels R, Granic I (2016). A neurofeedback video game (MindLight) to prevent anxiety in children: a randomized controlled trial. Comput Hum Behav.

[22] Merry SN, Stasiak K, Shepherd M, Frampton C, Fleming T, Lucassen MFG (2012). The effectiveness of SPARX, a computerised self help intervention for adolescents seeking help for depression: randomised controlled non-inferiority trial. BMJ.

[23] Kollins SH, DeLoss DJ, Cañadas E (2020). A novel digital intervention for actively reducing severity of paediatric ADHD (STARS-ADHD): a randomised controlled trial. Lancet Digit Health.

[24] Smith H, Budworth L, Grindey C (2022). Co-production practice and future research priorities in United Kingdom-funded applied health research: a scoping review. Health Res Policy Sys.

[25] White BM, Prasad R, Ammar N, Yaun JA, Shaban-Nejad A (2024). Digital health innovations for screening and mitigating mental health impacts of adverse childhood experiences: narrative review. JMIR Pediatr Parent.

[26] Chinsen A, Berg A, Nielsen S (2025). Co-design methodologies to develop mental health interventions with young people: a systematic review. Lancet Child Adolesc Health.

[27] Green T, Bonner A, Teleni L (2020). Use and reporting of experience-based codesign studies in the healthcare setting: a systematic review. BMJ Qual Saf.

[28] Duffy A, Boroumandzad N, Sherman AL, Christie G, Riadi I, Moreno S (2025). Examining challenges to co-design digital health interventions with end users: systematic review. J Med Internet Res.

[29] Skivington K, Matthews L, Simpson SA (2021). A new framework for developing and evaluating complex interventions: update of Medical Research Council guidance. BMJ.

[30] Donetto S, Pierri P, Tsianakas V, Robert G (2015). Experience-based co-design and healthcare improvement: realizing participatory design in the public sector. Des J.

[31] Bate P, Robert G (2006). Experience-based design: from redesigning the system around the patient to co-designing services with the patient. Qual Saf Health Care.

[32] Dingsøyr T, Nerur S, Balijepally V, Moe NB (2012). A decade of agile methodologies: towards explaining agile software development. J Syst Software.

[33] Khaled R, Vasalou A (2014). Bridging serious games and participatory design. Int J Child Comput Interact.

[34] Kocalevent RD, Hinz A, Brähler E (2013). Standardization of the depression screener Patient Health Questionnaire (PHQ-9) in the general population. Gen Hosp Psychiatry.

[35] Löwe B, Decker O, Müller S (2008). Validation and standardization of the Generalized Anxiety Disorder Screener (GAD-7) in the general population. Med Care.

[36] Perrin S, Meiser-Stedman R, Smith P (2005). The Children’s Revised Impact of Event Scale (CRIES): validity as a screening instrument for PTSD. Behav Cogn Psychother.

[37] Russell D, Peplau LA, Cutrona CE (1980). The revised UCLA Loneliness Scale: concurrent and discriminant validity evidence. J Pers Soc Psychol.

[38] Stewart-Brown S, Tennant A, Tennant R, Platt S, Parkinson J, Weich S (2009). Internal construct validity of the Warwick-Edinburgh Mental Well-Being Scale (WEMWBS): a Rasch analysis using data from the Scottish Health Education Population Survey. Health Qual Life Outcomes.

[39] Kaufman EA, Xia M, Fosco G, Yaptangco M, Skidmore CR, Crowell SE (2016). The Difficulties in Emotion Regulation Scale Short Form (DERS-SF): validation and replication in adolescent and adult samples. J Psychopathol Behav Assess.

[40] Staniszewska S, Brett J, Simera I (2017). GRIPP2 reporting checklists: tools to improve reporting of patient and public involvement in research. BMJ.

[41] Nagata JM, Imbago-Jácome D, Choonara S (2025). Reporting of research with adolescent and youth engagement. Lancet Child Adolesc Health.

[42] Butcher I, Williams AM, Hugh-Jones S (2023). A participatory action research study to explore adolescents’ experiences of adverse childhood experiences (ACEs) through creative workshops: a protocol. medRxiv.

[43] Pethybridge R, Smith L (2024). Embodying care: trauma-informed practice in community dance. J Appl Arts Health.

[44] Reay E, Ma M, Mankee-Williams A, Pavarini G, Shaughnessy N, Bhui K (2024). Stones in our pockets: mental health dimensions of grief in contemporary video games. medRxiv.

[45] Zhu H, Ma M, Sansoy H, Butcher I, Bisal N, Bhui K, Thomas A, Meyer M, Zank M (2026). Serious Games.

[46] Robjant K, Fazel M (2010). The emerging evidence for narrative exposure therapy: a review. Clin Psychol Rev.

[47] Nadeau JW (2006). Metaphorically speaking: the use of metaphors in grief therapy. Illness Crisis Loss.

[48] Goldberg RM, Stephenson JB (2016). Staying with the metaphor: applying reality therapy’s use of metaphors to grief counseling. J Creativity Ment Health.

[49] Reay E (2024). The Child in Videogames: From the Meek, to the Mighty, to the Monstrous.

[50] Fazel M, Stratford HJ, Rowsell E, Chan C, Griffiths H, Robjant K (2020). Five applications of narrative exposure therapy for children and adolescents presenting with post-traumatic stress disorders. Front Psychiatry.

[51] Lely JCG, Smid GE, Jongedijk RA, W. Knipscheer J, Kleber RJ (2019). The effectiveness of narrative exposure therapy: a review, meta-analysis and meta-regression analysis. Eur J Psychotraumatol.

[52] Schauer M, Neuner F, Elbert T (2017). Evidence-Based Treatments for Trauma Related Disorders in Children and Adolescents.

[53] Pavarini G, Smith LM, Shaughnessy N (2021). Ethical issues in participatory arts methods for young people with adverse childhood experiences. Health Expectations.

[54] Pavarini G, Reay E, Crowe E (2024). Ethical implications of digital gaming interventions for mental health: systematic review and critical appraisal. OSF.

[55] Stieler-Hunt C, Jones CM, Rolfe B, Pozzebon K (2014). Examining key design decisions involved in developing a serious game for child sexual abuse prevention. Front Psychol.

[56] da Silva TS, Silveira MS, Maurer F, Hellmann T (2012). User experience design and agile development: from theory to practice. J Software Eng Appl.

[57] Cross S, Mangelsdorf S, Valentine L (2025). Insights from fifteen years of real-world development, testing and implementation of youth digital mental health interventions. Internet Interventions.

[58] Verschueren S, Buffel C, Vander Stichele G (2019). Developing theory-driven, evidence-based serious games for health: framework based on research community insights. JMIR Serious Games.

[59] Kochanowska M, Gagliardi WR, Raposo D, Neves J, Silva J (2022). Perspectives on Design II: Research, Education and Practice.

[60] Randazzo C, Scott CF, Bellini R Trauma-informed design: a collaborative approach to building safer online spaces.

[61] Spahl W, Motta V, Woodcock K, Rubeis G (2024). Gamified digital mental health interventions for young people: scoping review of ethical aspects during development and implementation. JMIR Serious Games.

[62] Danilovic S (2018). Game design therapoetics: autopathographical game authorship as self-care, self-understanding, and therapy. http://hdl.handle.net/1807/89836.

[63] Dewhirst A, Laugharne R, Shankar R (2022). Therapeutic use of serious games in mental health: scoping review. BJPsych Open.

