# Supplementary material for: The ACE of Hearts Serious Game for Young People With Adverse Childhood Experiences: Experience-Based Co-Design and Agile Development Study
Source: JMIR Serious Games. 2026 Oct 5;14:e99789. doi: 10.2196/99789 (PMC13638077; doi:10.2196/99789)
Supplement: Supplementary file 1 [file games-v14-e99789-s001.docx]

# Multimedia Appendix: Supplementary tables and figures

## Table S1. Summary of Co-Design Sessions, Stakeholders, and Activities

| **Session** | **Date** | **Aim** | **Participants** | **Format** |
| --- | --- | --- | --- | --- |
| 1 | Jun 2022 | Game concept and preference exploration with young people | 13 YP | Hybrid |
| 2 | Oct 2023 | Continued concept development and storyline ideation | YP + research team | Hybrid |
| 3 | Nov 2023 | Playtesting and feedback on Horse & Foal | YP + dev team | Zoom |
| 4 | Dec 2023 | Playtesting and feedback on Hard Times (poverty game) | YP + dev team | Zoom |
| 5 | Jan 2024 | Embedded ethics review session | YP + stakeholders | Hybrid |
| 6 | Jan 2024 | Game feedback and iterative refinement | YP + dev team | Hybrid |
| 7 | Feb 2024 | Embedded ethics review session | YP + stakeholders | Hybrid |
| 8 | Feb 2024 | Co-design with adult stakeholders and young people on all four mini-games | 9 adult stakeholders + 6 YP | Zoom / Miro |
| 9 | Apr 2024 | NET game (Dial It Back) co-design and playtesting | YP + clinical advisors | Hybrid |
| 10 | Oct 2024 | Final game feedback and refinement | YP + dev team | Hybrid |

## Table S2. Sociodemographic Characteristics of Co-Design Participants

*Note:* 18 young people participated in co-design; survey data available for n=14 participants who completed the baseline questionnaire.

| **Variable** | **Value (n=14)** |
| --- | --- |
| **Age (years), M (SD)** | 19.1 (3.4) |
| Range | 11–24 |
| **Gender, n (%)** |  |
| Man/Boy | 8 (57.1) |
| Woman/Girl | 3 (21.4) |
| Non-Binary | 1 (7.1) |
| Other | 2 (14.3) |
| **Trans Status, n (%)** |  |
| Yes | 3 (21.4) |
| No | 11 (78.6) |
| **Ethnicity, n (%)** |  |
| English, Welsh, Scottish, NI, British | 6 (42.9) |
| Other White background | 3 (21.4) |
| Caribbean | 1 (7.1) |
| Other Black, African or Caribbean | 1 (7.1) |
| Mixed/Multiple Ethnic Background | 1 (7.1) |
| Mixed, White and Black African | 1 (7.1) |
| Other ethnic group | 1 (7.1) |
| **Sexuality, n (%)** |  |
| Heterosexual/straight | 2 (14.3) |
| Bisexual or pansexual | 6 (42.9) |
| Prefer not to say | 2 (14.3) |
| Other | 4 (28.6) |
| **Religion, n (%)** |  |
| Christian | 5 (35.7) |
| Muslim | 3 (21.4) |
| Buddhist | 1 (7.1) |
| Baha'i | 1 (7.1) |
| No religion/Agnostic/Unsure | 4 (28.6) |
| **Born in the UK, n (%)** |  |
| Yes | 10 (71.4) |
| No | 4 (28.6) |
| **Education Status, n (%)** |  |
| School/Sixth Form College | 1 (7.1) |
| Higher education (University) | 10 (71.4) |
| Further education | 1 (7.1) |
| Not in education | 2 (14.3) |
| **Employment Status, n (%)** |  |
| Full-Time | 2 (14.3) |
| Part-Time | 4 (28.6) |
| Zero-hour contract/holiday job | 5 (35.7) |
| Not employed | 2 (14.3) |
| Missing | 1 (7.1) |
| **Hunger Status, n (%)** |  |
| Not at all | 13 (92.9) |
| Sometimes/Once or Twice | 1 (7.1) |
| **Parents/Carers born in the UK, n (%)** |  |
| Yes, both parents | 6 (42.9) |
| Yes, one parent | 6 (42.9) |
| No | 2 (14.3) |
| **Parent/Caregiver Status, n (%)** |  |
| Yes, more than one parent/caregiver | 12 (85.7) |
| Yes, one parent/caregiver | 2 (14.3) |
| **Foster/Residential Care Status, n (%)** |  |
| No | 14 (100.0) |
| **Location Type, n (%)** |  |
| Urban | 9 (64.3) |
| Rural/Coastal | 5 (35.7) |
| **Parent/Carer Education Status, n (%)** |  |
| Not sure/Other | 2 (14.3) |
| Attended secondary school | 1 (7.1) |
| AS/A2 Level | 2 (14.3) |
| Technical/Vocational Training | 4 (28.6) |
| Bachelor's Degree | 4 (28.6) |
| Master's Degree | 1 (7.1) |

## Table S3. Mental Health Characteristics of Co-Design Participants

| **Clinical Variable** | **Value (n=14)** |
| --- | --- |
| **Neurodivergence, n (%)** |  |
| ADHD/ADD or Attention Differences | 4 (28.6) |
| Autism or Autism Spectrum Condition | 7 (50.0) |
| Demand Avoidance | 2 (14.3) |
| Dyscalculia | 0 (0.0) |
| Dyslexia | 2 (14.3) |
| Dyspraxia | 0 (0.0) |
| Intellectual/Learning Disability | 3 (21.4) |
| Selective/Situational Mutism | 0 (0.0) |
| Language Impairment | 2 (14.3) |
| Synaesthesia | 0 (0.0) |
| Tourette's Syndrome | 0 (0.0) |
| **Number of ND Conditions, n (%)** |  |
| None | 4 (28.6) |
| 1 condition | 6 (42.9) |
| 2 conditions | 2 (14.3) |
| 3+ conditions | 2 (14.3) |
| **Depression Severity (PHQ-9), M (SD)** | 8.8 (4.5) |
| No/Minimal Depression (0–4), n (%) | 3 (21.4) |
| Mild Depression (5–9), n (%) | 4 (28.6) |
| Moderate Depression (10–14), n (%) | 6 (42.9) |
| Moderate-Severe Depression (15–19), n (%) | 1 (7.1) |
| Severe Depression (20–27), n (%) | 0 (0.0) |
| **Anxiety Severity (GAD-7), M (SD)** | 8.4 (3.9) |
| No/Minimal Anxiety (0–4), n (%) | 1 (7.1) |
| Mild Anxiety (5–9), n (%) | 9 (64.3) |
| Moderate Anxiety (10–14), n (%) | 2 (14.3) |
| Severe Anxiety (15–21), n (%) | 2 (14.3) |
| **Mental Wellbeing (SWEMWBS), M (SD)** | 22.6 (4.1) |
| **Loneliness (UCLA-4), M (SD)** | 9.4 (2.5) |
| No/Low Loneliness (4–6), n (%) | 2 (14.3) |
| Moderate Loneliness (7–9), n (%) | 5 (35.7) |
| Moderate-High Loneliness (10–12), n (%) | 5 (35.7) |
| Severe Loneliness (13–16), n (%) | 2 (14.3) |
| **Emotion Regulation (DERS-SF Total), M (SD)** | 50.1 (8.4) |
| DERS Awareness, M (SD) | 9.9 (3.1) |
| DERS Clarity, M (SD) | 8.1 (3.0) |
| DERS Goals, M (SD) | 10.3 (2.7) |
| DERS Impulse, M (SD) | 5.8 (3.2) |
| DERS Non-Acceptance, M (SD) | 9.1 (3.8) |
| DERS Strategies, M (SD) | 7.0 (1.8) |
| **Impact of Events – PTSD (CRIES-8), M (SD)ᵃ** | 22.5 (7.7) |
| Intrusion Symptom Severity, M (SD) | 9.2 (5.4) |
| Avoidance Symptom Severity, M (SD) | 11.7 (5.3) |

*ᵃ CRIES-8 data available for n=13.*

## Table S4. The "You Said, We Did" feedback and design decision log

| **Category** | **Feedback received** | **Response and action taken** |
| --- | --- | --- |
| **Feedback on the *Horse and Foal*** | | |
| **Narrative pacing and emotional tone** | **On sudden death and emotional impact:**   - “Shift to loss was too dramatic at the end” - “Ending was very sudden…it made me emotional because of how sudden it was. There’s a lot to unpack emotionally” - “I felt sad at the ending, didn't want to make the older horse leave the foal…ending depends on the purpose” - “Ending quite jarring but I think that helped to convey the loss” | - Additional chapter created to reflect processing grief and resolution, extending story length - New dialogues added to enhance visual hints and metaphor clarity - Implemented foreshadowing to reduce abruptness while maintaining emotional impact |
|  | **On “take-home message”:**   - “No matter what…the character really tried but nothing changed the outcome” - “Despite all best efforts she dies; you feel like even after all that you did it wasn’t enough” - Liked realism that “sometimes things occur just because” but worried it might discourage help-seeking - Concerns about implied hopelessness, e.g., “it’s goanna be hard, no matter how much help you get” | - Added supportive messages and explicit reassurance in-game - Included message of self-care, emphasizing external support resources |
|  | **Proposed solutions from co-researchers (young people, adult stakeholders and the research team):**   - “Add reassurances…within the game it should be clear that this was not her fault” - “Important reminder that it isn’t that person’s fault… ‘You did the best you could’” - Suggestion of “Grandpa Wispa returning and clarifying things” - Idea of “ghost of the foal coming to embrace the horse” to symbolize acceptance - “Use resources built up throughout the game for self-care” - Seasons metaphor suggested for cyclical nature of grief | - These suggestions have informed the final ‘grief’ chapter, e.g., new ending scene that integrates Grandpa Wispa and leaves the player with a hopeful note as suggested. |
| **Game feature, visual and arts styles** | - YP could not easily find the horse & foal game after the phone chat, potentially needs better transition | - Improved UX by clearly marking the transition from phone chat to the mini-game with interactive button prompts. |
|  | - Colour was too dark and cold | - Revised colour palette introducing brighter, seasonal backgrounds (spring/summer) to reduce bleak visuals |
| **Gameplay and engagement** | - Concerns about keeping attention, gameplay described as “slow” - Suggested clearer indication of playtime length at start | - Enhanced gameplay and interactive features |
| **Feedback on *Dial It Back*** | | |
| **Narrative and emotional tone** | **Clarity on trauma representation:**   - Clarity on friendships portrayal; questioned game’s assumption everyone has close friends - Found the home environment “too big and tidy” and questioned realism - Visual resource choices, e.g., bedding imagery and superhero mask metaphor unclear | - Clarified narrative to explicitly acknowledge multiple and unseen ACEs beyond physical trauma representation, e.g., neurodiversity - Adjusted visuals for clarity and appropriateness |
| **ACEs and trauma** | - Raised concern that visible physical trauma (car crash) overshadowed less visible ACEs - Positive response to mix of ACEs portrayed realistically, praised metaphor usage | - Continued explicit positive portrayal of overlapping ACE experiences with metaphorical depth maintained |
| **Feedback on *Out of My Shell*** | | |
| **Narrative, metaphor and lived experiences** | **Narrative and metaphor**:   - Metaphor praised as effective: “I love the metaphor, an inventive way preventing themes from being too upsetting” - Questions about clear portrayal of eating disorders in-game - Concerns about potential misinterpretation of dragons as destructive; suggested explicit narrative clarification. | - Expanded and clearly communicated dragon metaphor within the narrative to prevent misinterpretation |
|  | **Understanding experiences of gender dysphoria:**   - Requested clear communication that understanding dysphoria is challenging for those without personal experience - Suggested portrayal of community support and safe spaces in the narrative - Importance of highlighting multiple experiences of being trans, including detransition positively without stigma. | - Added explicit dialogue about experiences of dysphoria, clearly acknowledging complexity and diversity of trans experiences - Integrated feedback about portraying diverse trans experiences positively, including nuanced treatment of detransition narratives |
|  | **On “take-home message”:**   - Youth felt positively about optimistic/pessimistic balance, interactive nature, and supportive characters: “game definitely feels more positive” | - Emphasised explicitly positive, balanced emotional messaging (optimism and realism) throughout gameplay |
| **Gameplay and engagement** | - Suggested multiplayer or cooperative play to represent friendships/support networks explicitly | - Suggestion noted for potential future update to encourage peer support explicitly |
| **Feedback on *Hard Times*** | | |
| **Narrative and gameplay** | **Balance between realism and gameplay enjoyment**：   - Poverty topic felt heavy: “worried about trivialisation” - Suggested including practical coping strategies - Importance of highlighting systemic issues, “an unfair system designed to keep people just within margins” - Questions about target audience clarity (younger children vs teenagers) - Suggested linking content explicitly to realistic school situations to enhance empathy and inclusion of prejudice/racism themes explicitly (e.g., “landlord prejudice” and false accusations) | - Balanced realism with hopeful gameplay elements and practical support options - Clarified game’s educational and therapeutic aims for different age groups explicitly in the accompanying materials - Integrated explicit references to highlight social issues clearly (e.g., evicted from the house for not paying rent) |
|  | **On “take-home message”:**   - Wanted clarity on take-home messages, suggesting emphasis that poverty “is not your fault” | - Reinforced the narrative message that systemic factors cause poverty, explicitly stating “it's not your fault” |
| **Other general feedback** | | |
| **Cosy den room** | - Attendees were asked what chill out things would they like to see in the game: Journal, Calendar to show important dates and how long you have been playing for | - Cosy Den enhancements noted for possible future updates. |
| **Language and cultural references** | - Confusion about specific reference, e.g., size of Kansas, that participants unfamiliar or found odd - Colloquial language occasionally felt “awkward” or overly juvenile   • Suggestion for pop culture references, more relatable bands, shows, or films | - Revised language reference and replaced with universally recognisable metaphor. - Reduced colloquialisms to better match youth expectations and clarity |
| **Character design** | - Concern with protagonist described as “‘green-haired’ weirdo,” could reinforce negative mental health stereotypes - Preferred an “ordinary-looking” character to represent broader mental health issues | • Retained existing character design due to narrative importance but adjusted descriptions to remove negative connotations (“weird” dropped)  • Diversified representation in other characters across mini-games |

## Table S5. Summary of Narratives, Visual, Auditory, Game Mechanics, and Haptic Features

| **The games** | **Narrative** | **Visual** | **Audio** | **Mechanics** | **Haptic** |
| --- | --- | --- | --- | --- | --- |
| *Cozy Den*:  A central room where players can customise and interact with their own in game bedroom | Virtual room that serves as the player’s home base and no explicit storyline in the hub. The room is a space feel safe for the player. | Visual design is cozy and warm, depicting as a young person’s bedroom filled with furnishings and personal items. | Gentle soothing music.  Players can change the music by tapping the sound equipment.  Guitar strum when playing the guitar. | Campaign area: with desk computer and mobile to unlock the mini games by reading and sending messages with friends, and a storyboard to show game characters.  Customisation: players can customise their room, such as making bed, switching poster and choose their own music. | Pick up the mobile and press the message app to read the message and unlock the mini games.  Press buttons to customise and interact with various game mechanics.  Interactions such as play the guitar, drawing, feed the fish, and read the books. |
| *Horse and Foal*:  A 2D storytelling game about bereavement and parental responsibility being placed on a young person | Carla (the horse) takes on a parental role for her sister Rhea (the foal), ensuring her safety whilst navigating a series of obstacles and dilemmas | Story beats are presented with a Disney-esque art style and a series of sketchbook and diary entries. Pastoral fields with four seasons change to convey the four chapters of stories; black mist fog as metaphor of illness; animal characters (horse, foal, fox, crow) as metaphors of helpers. | Smooth music with bird’s sound occasionally; tense forest ambience with heartbeats in fear moments; fox cries and soothing sounds from helpers. | Black mist and anxiety: a metaphor representing younger sister Rhea’s terminal illness, chases the horse and foal. Rhea’s anxiety builds over time and the player needs keep moving forward while stop to reassure Rhea.  Crossroads: Each chapter ends at a crossroads where Carla faces a difficult dilemma between parental responsibility or her personal life. At the grief stage, there is only one option “keep moving on” to encourage Carla that its time to move on her own life.  Obstacles: Logs and rocks block the player’s path, a metaphor for life’s obstacles.  Animal characters (auntie fox, neighbour crow, momma bear, therapy raccoon and grandpa wisp): NPCs will offer the player a variety of items which will either help or hinder them. | Player needs to hold the “hoof” button to move forward but must stop and hold the “heart” icon to reduce anxiety.  Obstacle: Player must tap the screen to destroy the obstacles.  Inventory: Player can use items from the inventory for help.  Grief button: player needs to hold the “grief” button to reduce grief. |
| *Dial It Back*:  A 2D game inspired by Narrative Exposure Therapy (NET) about a young person living with a disability | Oli and Iris (eyefloator) journey back through Oli’s memories of the past ten years. Narrative exposure therapy  Oli recalls good and bad memories, and the player chooses to place flowers or stones down for them.  It covers his experiences with neurodiversity and finding others like him, and of a car accident and the struggles that followed. | In an ethereal landscape representative of memory – called Anamnesis.  Surreal memory world (starry night); friend guide character; diary entries on-screen; flowers and stones representing good and bad memories; constellations puzzles. | Ethereal music; supportive guide voice (Iris); memory-specific sound cues (owl hoot, glass break) for sensory recall; | Unlock the memories: find four detailed sensations from the memory: smell, sound, touch, and feeling (emotion).  Interactions: place flowers and stones to mark memory as good or bad; connect the stars for constellations; button slows things down. | Payer can move the eye glass around the brain with figure to find sensations and hold the “focus” button to recall.  Player can touch a star and drag finger to draw the constellations. Light vibration on finding each memory detail; intense shaking during trauma memory; slow breathing pulse during grounding exercise. |
| *Out of Shell*:  A 2D tower defence style game about a young person experiencing gender dysphoria | An exploration of a young trans person Oli growing up and struggling to accept their identity. It covers segments of stories, including the fear of being found out, some struggles with internalised transphobia as well as hearing external bigotry from other people, and slowly finding healthier ways to cope and explore identity in a safe environment. | Pixel art tower-defence game with dragon, eggs; dragon egg “shell” visual motif. | Smooth music and battle sound during the tower defence gameplay. | Flo’s dysphoria is represented through a metaphor of being a dragon in disguise.  Agnes and Leona are mystical creatures and represent mental health guardians.  The player is led through several challenges and being told new segments of a story, with gameplay of defending your “nest” (representing your mental health) at the end of each segment.  Throughout the game, the player will be led through mindfulness techniques, such as breathing exercises. | The player will have a “budget” of emotional points which they can use to pick different characters. |
| *Hard Times*:  A 3D first person game about a family struggling with finances | Mina lives in a rented flat in London with her mum and little brother. Times are tough and they’re struggling to make rent. Experiencing life in her shoes, the player has to make tough choices between money and basic wellbeing needs. | Scripted story events in a flat; user interface shows money count; visual choice prompts with immediate consequence cues. Mood: Players in-game mood changed to the shown emoji | Minimal background music; authentic sound effects (doors, city noise, coins); loud knocking and silence to emphasize dread. | Narrative adventure with exploration with 4 months of time duration; resource collection (scavenge money, items); facing choice dilemmas (e.g. buy food vs save money); time progression morning to night; multiple endings (rent partially paid vs eviction notice). | Player can move, pick up, inspect and throw/break (e.g. the piggy bank) items |

**Table S6. Ethical considerations and design decisions on safeguarding and ethics.**

| **Questions related to ethical dimensions** | **Feedback received** | **Response and action taken** |
| --- | --- | --- |
| **Representation:**  1. Does the game represent the realities of young people across different age groups, regions,  rural/urban areas and including ethnic minorities, those who are neurodivergent, refugees  and LGBTQIA+? | - Concerns around stereotyping characters: “Green-haired weirdo” might stigmatise mental health issues - Participants asked explicitly for ethnic/cultural markers in-game narratives | - Diversified characters’ appearance and backgrounds across games - Enhanced culturally accurate elements in game narratives (e.g., cosy den customisation, immigrates background in Hard Times) |
| **Accessibility (technical):** 2. Is the game accessible to young people? (in terms of format and technical requirements e.g.,  required bandwidth)? | - Gameplay only includes mobile version; older or other low-spec devices being excluded - Difficulty reading small text, especially for visually impaired players | - Compatibility with older and low-spec devices (Apple Store, Android Play Store) - Improved font size |
| **Accessibility (content):**  3. Is the content of the game (e.g., visuals, story) accessible to young people? | - Dark colour palettes described as gloomy - Game narrative occasionally hard to follow, metaphor use unclear | - Updated visuals with alternative colour palettes |
| **Agency:** 4. Does the game provide young people with opportunities for personalisation and control  over the game environment? | - Participants felt frustrated when game outcomes couldn’t be influenced by choices - Players requested options to personalise or have visible impact | - Clarified narrative intent: some stories reflect uncontrollable life events, yet integrated explicit reassurance in dialogue - Personalisation options in Cosy Den |
| **Privacy and transparency:** 5. Is privacy respected, and information transparency maintained? 6. Is it clear to the young people who made the game  and how it is being managed? 7. Is it clear what the game does and doesn’t do, potential positives and negatives? | - Youth asked clearly about data handling - Co-design participants asked game’s intentions, requesting clear communication about developers, purpose, and commercial interests - Concerns around potential trivialisation of serious mental health issues - Clear indication needed about the limits of game’s therapeutic value | - Provided explicit in-game information about data use and privacy protection measures - Explicit communication provided during the co-design process - Clarified that the game is intended for supportive and educational purposes, not clinical intervention |
| **Emotional safety:** 8. Is emotional content presented responsibly to avoid triggering players?  9. Does the game connect young people to supportive or external resources?  10. Do young people know who to contact if they have an issue with the game or need support? | - Abrupt, intense emotional content caused distress - Participants requested in-game signposting to external help - feedback received on needing guidance about how to report issues or seek help | - Extended narrative epilogues to process grief and emotions - Implemented foreshadowing, supportive dialogues, and reassurance messages - Added in-game resources and links to support organisations - Made signposting and external resource more obvious |
| **Positive motivation:** 11. Does the game motivate users in a positive way and balanced play to prevent overuse or addiction? | - Participants suggested to provide self-care and play time reminders | - Integrated self-care reminders (e.g., mindfulness exercises in-game) |
| **Impact:**  12. Does the game encourage open dialogue about difficult experiences and support seeking?  13. Does the game address mental health and ACEs in a responsible, non-stigmatising and non-  trivialising way? | - Concerns expressed about potential trivialisation or harmful portrayals of ACEs and mental health (poverty, dysphoria, trauma) - Participants requested portrayals that avoid stereotypes and stigmas | - Revised and clarified narratives and dialogues to responsibly portray mental health and ACEs with explicit sensitivity and nuance - Avoided stereotypes, and used diverse, respectful representations (e.g., portrayal of dysphoria experiences and responsible metaphor use) |

**Figure S1.** Favourite games (top) and factors rated as important for the gaming experience (bottom), reported by 13 young people (aged 12-24 years) during the concept exploration workshop of the ACE of Hearts co-design study, United Kingdom, June 2022.
